# Axial rod slip at the end-of-construct screw in scoliosis surgery: relevance, occurrence and prevention

**DOI:** 10.1007/s43390-024-00925-9

**Published:** 2024-08-20

**Authors:** T. P. Schlösser, I. Blaauw, M. R. van der Valk, Guido van Solinge, C. Faber, M. C. Kruyt

**Affiliations:** 1 Department of Orthopedic Surgery, University Medical Center Utrecht, P. O. Box 85500, 3508 Utrecht, GA The Netherlands; 2 Department of Orthopedics UMC Groningen, Groningen, The Netherlands; 3 Department of Orthopedics Isala, Zwolle, The Netherlands

**Keywords:** Pedicle screw, Adolescent idiopathic scoliosis, Mesa 2 deformity, Slip

## Abstract

**Purpose:**

Despite standardized biomechanical tests for spinal implants, we recently recognized pedicle screw failure to maintain the rod fixated as a clinical concern in scoliosis surgery. This occurrence study investigates the risk and magnitude of axial rod slip (ARS), its relation with technique and preventive measures.

**Methods:**

Retrospective multicenter review of all primary scoliosis cases (2018–2020) with > 1 year FU from three centers, instrumented with uniplanar screws and 5.5 mm CoCr rods (Mesa 2, Stryker Corporation, Kalamazoo, MI, USA). ARS was defined as > 1 mm change in residual distal rod length from the screw in the lowest instrumented vertebra (LIV) and assessed by two independent observers. Slip distance, direction, relation to distal screw density and time of observation were recorded, as well as the effect of ARS on caudal curve increase. To prevent slip, more recent patients were instrumented with a different end-of-construct screw (Reline, NuVasive Inc. San Diego, CA, USA) and analyzed for comparison.

**Results:**

ARS risk was 27% (56/205) with a distance of 3.6 ± 2.2 mm, predominantly convex. 42% occurred before 4 months, the rest before 1 year. The caudal curve substantially increased three times more often in patients with ARS. Interobserver reliability was high and slip was in the expected direction. ARS was unrelated to distal screw density. Remarkable variation in ARS rates (53%, 31%, 13%) existed between the centers, while there was no difference in mean screw density (≈1.3 screws/level) or curve correction (≈60%). Revision surgery for ARS was required in 2.9% (6/207). Using the different end-of-construct screw, ARS risk was only 2% (1/56) and no revisions were required.

**Conclusion:**

This study demonstrates the prevalence of axial rod slip at the end of construct in scoliosis surgery and its clinical relevance. While minimal ARS can be subclinical, ARS should not be mistaken for adding on. The most severe ARS predominantly occurred convex at the high-loaded distal screw when L3 was the LIV. Longer constructs (LIV L3 or L4) have a higher risk of ARS. The minimal risk of ARS with another end-of-construct screw underscores the influence of screw type on ARS occurrence in our series. Further research is essential to refine techniques and enhance patient outcomes.

## Introduction

Surgical correction and stabilization with posterior spinal fusion of adolescent idiopathic scoliosis (AIS) is a well-accepted procedure that has been performed for more than 50 years now. Although the results were remarkably good from the beginning, the procedure has evolved from a bipolar concept with posterior hook anchors, as introduced by Harrington, to segmental instrumentation by Cotrel–Dubousset [1, 2]. As a consequence, the correction itself has improved tremendously which enabled the possibility to save (caudal) segments and perform selective thoracic fusions. The game-changing instrument to achieve these advancements was the pedicle screw [3, 4]. This screw provides superior fixation and allows stronger three-dimensional correction that is maintained during osseous fusion the next 3–12 months [5, 6]. We all expect that these advanced corrections will translate into better outcomes, especially on the long term with respect to sagittal balance and less fused segments [7, 8].

**Fig. 1:**
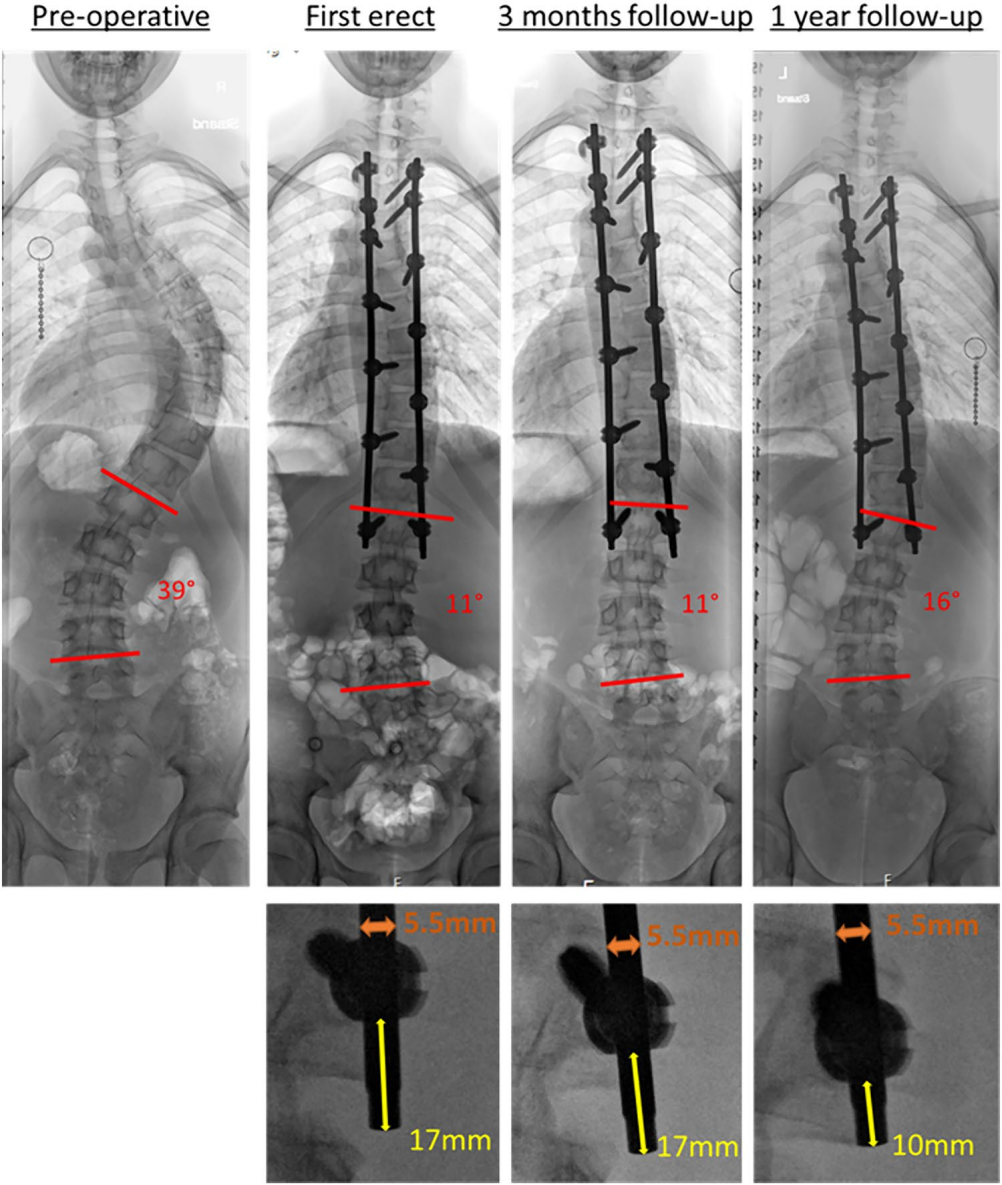
Example of a 15 year old boy with Lenke type 1A idiopathic scoliosis that underwent a spinal fusion from T3 to L1. Axial rod slip occurred between 3 and 12 months. As a consequence, the postoperative caudal curve increased 5°. Note that the caudal anchor consists of a single screw on one side and two screws on the other. Residual rod length measurement and the calibration on the rod are shown in the magnifications

In our academic pediatric spine unit, we apply a strategy since 2014 that involves low implant density, a dual-rod axial derotation maneuver and the ambition to keep the lower lumbar segments mobile. In a comparative study, this strategy compares favorable or equal to other ‘scoliosis schools’ in terms of 3D correction of the primary curves [9]. After more than 500 cases, however, we noticed an unusual failure of rod dislodgement out of a caudal end-of-construct screw. We attributed this to a technical failure, but soon after experienced a similar case. These two complications triggered us to look more closely at what is happening at the end of the construct and we noticed that in a considerable amount of patients, the rod has slipped a few millimeters at the lowest instrumented vertebra (LIV) (Fig. 1). In retrospect, this phenomenon appeared to be present for many years and several times, was the cause of what we recognized falsely as adding on (which is below and not within the construct). We first attributed this axial slip of the screw at the LIV in relation to the rod (ARS) to a technical/instrumentational problem, checked all instruments for accuracy, replaced them, and looked for signs of overloading the screws such as pull out or breakage. We also reviewed the English literature and the FDA Manufacturer and User Facility Device Experience database (MAUDE) which revealed that axial rod slip is very seldom reported and occurs with all top-loading screws [10]. As these investigations did not explain our findings, we decided to temporary change to another end-of-construct screw until we identified the problem. To understand whether the ARS was specifically related to our surgical technique or is a much broader phenomenon, we analyzed the occurrence of rod slip also in two other spine centers in the same country that used the same screw system for deformity surgery.

The aim of the current multicenter quality study is to primarily investigate the occurrence and magnitude of ARS at the LIV in posterior spinal fusion for AIS instrumented with the Mesa2 deformity system (Stryker Corporation, Kalamazoo, MI, USA), to determine its relevance in terms of curve progression or revision surgery, and to evaluate the relation to specific patient and instrumentation strategy related conditions. Furthermore, we had the opportunity to compare the results directly to an alternative end-of-construct screw (Reline, Nuvasive Inc., San Diego, CA, USA), as nothing else was changed in the procedure.

## Materials and methods

### Study design and population

**Fig. 2:**
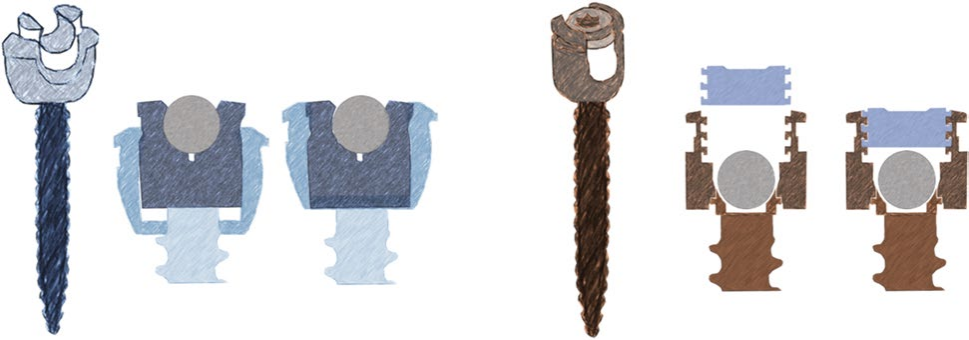
Screw locking principles are illustrated for both screws. Left: the Mesa2 deformity screw and rod fixation mechanism, designed to accomplish correction maneuvers in all planes with a very low profile and “Zero Torque technology^®^”. With final locking, the outer collet is retracted around the inner part that reaches above the equator of the rod. Right: the Reline top-loading screw with the “Helical Flange^®^ technology” set screw, an interlocking mechanism to minimize radial head splay. The set screw is inserted with a torque limiter

Due to the observed failures, this retrospective, multicenter occurrence study was initiated as a quality survey which is exempted from informed consent under national law. We discussed the option of receiving informed consent from all patients post hoc, but considered the impact too high for the  > 500 patients to learn that their spinal instrumentation may be failing, without extensive counseling. In accordance with the privacy rules, data were pseudonymized for the analyses. All consecutive patients that underwent posterior spinal fusion for scoliosis in one of the three participating centers between 2018 and 2020 were reviewed. The six scoliosis surgeons in these centers used the specific pedicle screw system for more than 5 years. All consecutive patients that met the following criteria were included:

- Ambulant patients < 25 years at the time of surgery, with adolescent idiopathic or idiopathic-like (with similar biomechanical loading and curve pattern, e.g., 22q11syndrome) scoliosis
- Treated with posterior-only spinal fusion and scoliosis correction, without three-column osteotomies.
- Treated with ∅5.5 mm Mesa2 Deformity Spinal System, using uniplanar pedicle screws at the LIV (Fig. 2).
- High-quality, biplanar, full-spine radiographs in the standing position obtained preoperative and at a minimum of two postoperative visits, with a minimum of 1 year follow-up.
- In case of ARS, the rod had to be positioned adequately on the first post-op image. This means to extent from the screw head at least 2 mm, on top of the hexagonal end (if present).

All patients from center 1 and 2 that fulfilled the same criteria and were operated after the surgical strategy was changed to the use of the alternative end-of-construct screws and had a minimum of 1 year radiographic follow-up were also included (Fig. 2).

### Surgical techniques

In all three centers, posterior instrumentation was in accordance with the level selection rules as described by Lenke et al. [11]. The exact surgical technique including the level of the lower instrumented vertebra (LIV) and the decision to do a selective fusion was at the discretion of the surgeons. After inferior facetectomies and strategic Ponte osteotomies, uniplanar pedicle screws (Mesa2 deformity system, Stryker, US) were inserted free hand with radiological support, and CoCr rods were the preferred rod material. All three centers used low-density constructs, and the primary correction maneuver was axial derotation with dual-rod correction. While center 1 and 2 generally instrumented the LIV and LIV-1 with three screws, in center 3, the distal anchor generally consisted of four screws. Furthermore, the surgeons in center 1 and 2 used a single-hinge distractor for final adjustment of LIV tilt, while in center 3, a less strong double-hinged parallel distractor was used. Local bone with or without supplemental allograft was used. After surgery, the patient was allowed to mobilize without a brace, but was informed to refrain from high impact activities for at least 6 months. Since 2021, in center 1 and 2, practice changed to the use of another end-of-construct screw (Reline, Nuvasive Inc., San Diego, CA, USA), while the rest of the procedure including screw density and correction strategy remained the same. Routine full-spine standing radiographs were acquired postoperatively, after 3 months and after 1 year with stitched posterior–anterior and lateral radiographs at center 1 and 2 and with biplanar radiography in center 3.

### Data collection

**Fig. 3:**
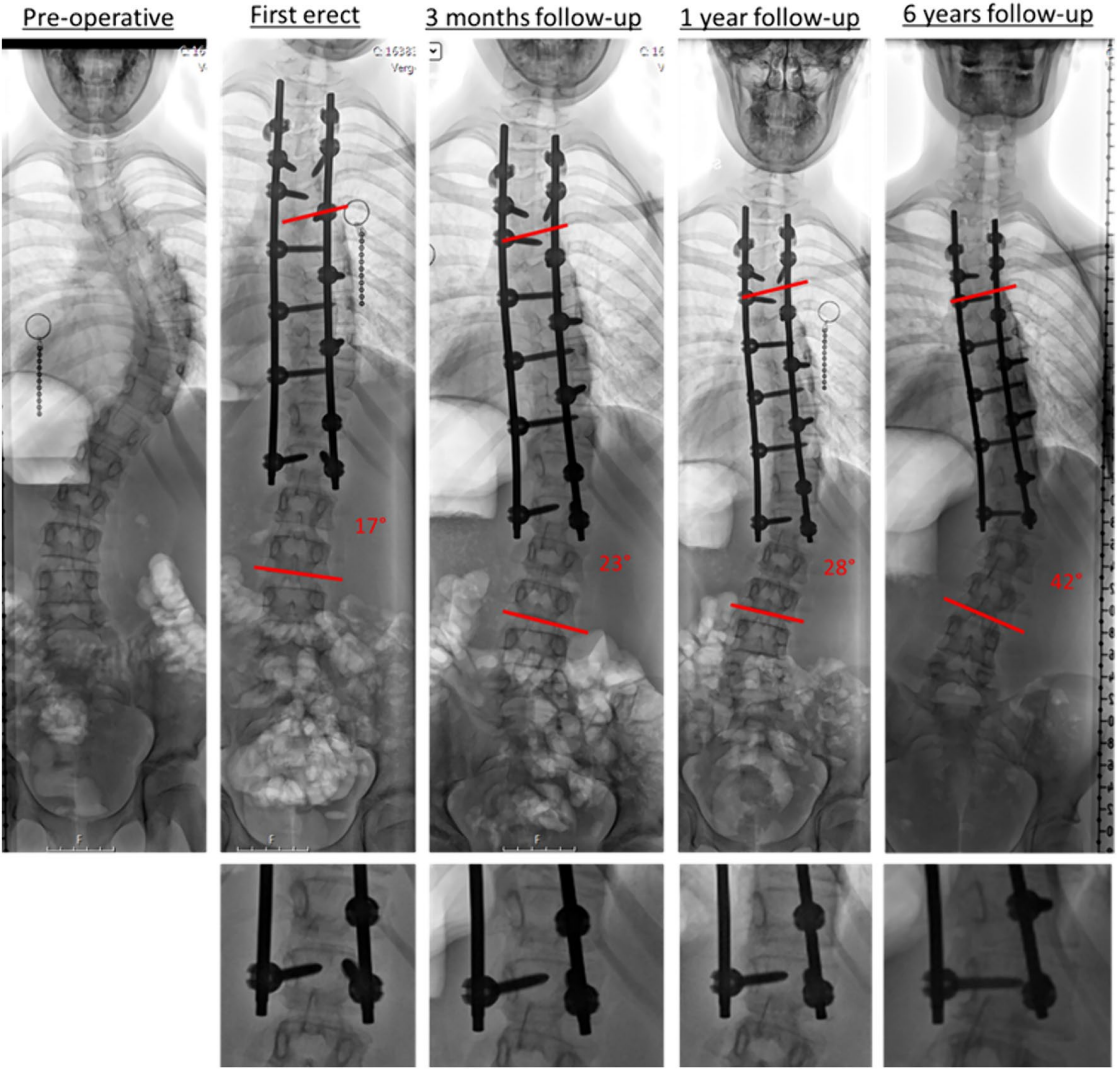
Example of a 13 year old girl with Lenke type 1A scoliosis that underwent a posterior spinal fusion from T3 to L1, with axial rod slip occurring after 3 months postoperative with progressive angulation that was initially regarded as adding on

The patient record was reviewed for curve etiology, previous surgery, weight, and age at index surgery. Furthermore, data were collected involving unplanned revision spine surgeries (UPROR) or indications for revision after index surgery, specifically if this involved distal instrumentation failure like rod displacement or adding on. Last preoperative and postoperative radiographs were assessed at each clinic by two trained independent observers (orthopedic resident and medical student with completed clinical rotation in spinal surgery) using the local picture archiving and communication systems. First, curve correction, screw density, and signs of screw loosening or breakage were analyzed. If a caudal (thoraco)lumbar curve was included (Lenke type 3–6), we normalized the LIV level for the amount of lumbar vertebrae by considering the most distal lumbar vertebrae always as L5. At 1 year follow-up, the presence of a curve progression > 5° of the distal segment including the LIV as compared to the first postoperative radiographs was determined (Figs. 1 and 3).

**Fig. 4:**
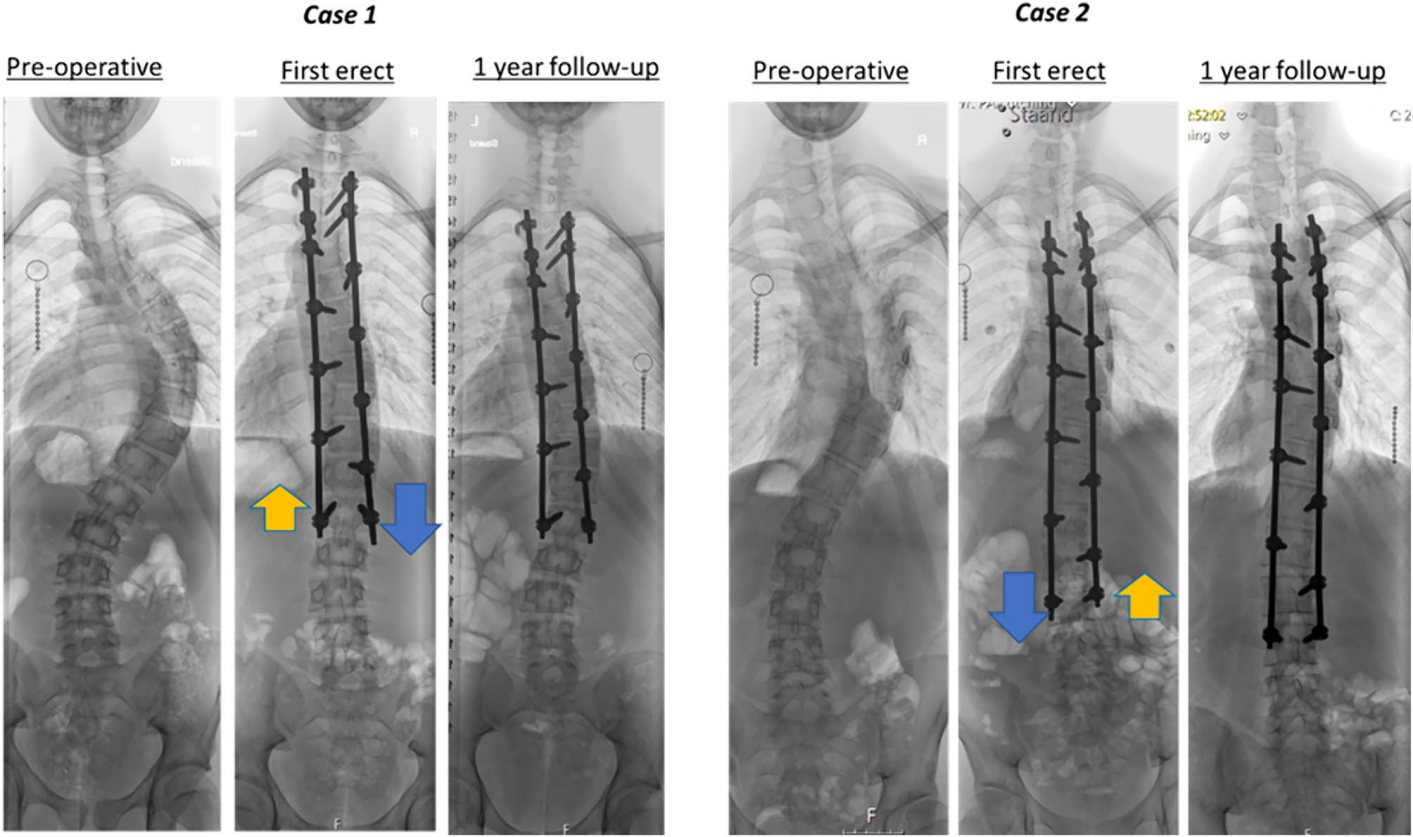
The arrows in case 1 and 2 illustrate the expected direction of rod slip. Case 1 idiopathic scoliosis with a relatively short fusion, and case 2 idiopathic-like syndromic scoliosis with a fusion to L3. Case 1 followed the expected direction on the convexity, whereas case 2 had a bilateral slip where the concavity did not follow the expected direction

To determine rod slip, the radiographs were magnified to visually detect a time-related change in residual rod length caudal to the LIV. Presence of ARS was defined as residual rod length change of  > 1 mm (calibrated on the 5.5 mm circular rod) between any of the postoperative radiographs (Fig. 1). Cases of ARS were reconfirmed and discrepancies resolved by a third observer, one of the spinal surgeons. The amount and direction of slip was measured as well as whether there was a screw at the level cranial to the LIV on the side of the slip. Based on the distal configuration, the *observed* direction of slip was related to the *expected* local forces, i.e., distraction on the convex side and compression on the concavity (Fig. 4). For assessment of interobserver reliability for slip magnitude, the two observers independently measured all 56 ASR cases.

### Statistics

Descriptive statistics were used to describe frequency and magnitude of ARS and revisions. To exclude a measurement error, the concordance between expected and observed direction of slip was calculated. Intraclass correlation coefficients and 95% confidence intervals were calculated (two-way random effects, absolute agreement, multiple raters). As the data on potential risk factors are obvious, additional post hoc t tests were considered not contributive.

##  Results

Demographics are given in Table 1. A total of 205 eligible cases treated with the standard screw were selected and analyzed 2–4 years after index surgery. The cohort with the alternative end-of-construct screw consisted of 56 patients and was analyzed 1–3 years after surgery. For the standard screw cases, there were no obvious differences between the clinics in terms of patient characteristics, curve correction (≈60%), and screw density (≈1.3). The only remarkable difference was the proportion of caudal curve instrumentations (LIV L3 or L4) which was considerably lower in center 3 (which had the least ARS), 15% compared to ≈ 47% in the other centers.

**Table 1:** Demographics of the standard cohort (with Mesa 2 deformity) and the alternative (with Reline) end-of-construct screw

	Center 1	Center 2	Center 3	Total
Standard				
* n* patients	87	34	84	205
Age at time of surgery in years	15.3	16.1	15.6	15.7
Weight at surgery in kg	57	63	57	585
BMI kg/m^2^	20.4	21.7	19.9	
Pre-operative Cobb angle major curve in °	62	56	63	61
Main curve correction as %	59	52	62	59
Screw density	1.26	1.32	1.34	1.30
Alternative end-of-construct screw				
* n* patients	39	17		56
Age in years	15.7	16.4		15.9

Screw density was calculated as the mean number of screws or hooks per level

The intraclass correlation coefficient for interobserver reliability and corresponding 95% confidence interval was 0.97 (0.96–0.98) for absolute measurement of rod slip. Axial rod slip occurred in 56 of 205 cases (27%), at a remarkably different rate and magnitude between the centers: 31, 53, and 13%, respectively. In comparison, in the entire alternative end-of-construct screw cohort, one slip was observed that occurred after 1 year (2%). In the standard screw cohort, ARS was a reason for revision surgery due to complete dislodgement or severe distal angulation in 6 patients (3%). This makes rod slip accountable for 21% of the indications for reoperation. Within this cohort, substantial progression of the caudal curve was found in 20% of the patients, three times more often in the patients that had ARS (38%) than in the patients without ARS (13%). As expected, this was different between the centers, with no clear effect of ARS in center 3. Distal instrumentation failure other than slip, e.g., loosening or broken screws, was found in 8 cases (4%) and the total amount of unplanned return to OR for any reason other than slip (e.g., infection) was 23 (11%). For the alternative screw cohort, 2 broken screws were observed and 1 UPROR (Table 2).

**Table 2:** Axial rod slip rates, and radiographic and clinical outcomes for all patients within the cohort

	Center 1	Center 2	Center 3	Total
Standard screw				
Patients with axial rod slip, n	27 (31%)	18 (53%)	11 (13%)	56 (27%)
Patients with axial rod slip per LIV				
T11	0 (0%)	1 (100%)	1 (9%)	2 (13%)
T12	6 (40%)	1 (13%)	2 (7%)	7 (18%)
L1	3 (20%)	2 (40%)	4 (20%)	9 (23%)
L2	0 (0%)	1 (33%)	1 (17%)	2 (17%)
L3	17 (47%)	9 (90%)	0 (0%)	26 (50%)
L4	1 (17%)	4 (80%)	3 (43%)	8 (44%)
UPROR	11 (13%)	9 (26%)	9 (10%)	29 (14%)
Patients with UPROR due to slip	5 (6%)	1 (3%)	0 (0%)	6 (3%)
Implant failures at LIV other than slip (%)	1 (1%)	4 (10%)	3 (3%)	8 (4%)
> 5° Curve progression	21 (23%)	14 (38%)	7 (8%)	42 (20%)
> 5° Curve progression in patients with axial rod slip	12 (44%)	8 (44%)	1 (9%)	21 (38%)
> 5° Curve progression in patients without axial rod slip	9 (15%)	5 (30%)	6 (8%)	20 (13%)
Alternative end-of-construct screw				
Patients with slip (% shown for total number of patients)	1 (2%)	0 (0%)	–	1 (2%)
UPROR	0 (0%)	1 (6%)	–	1 (2%)
Patients with UPROR due to slip	0 (0%)	0 (0%)	–	0 (0%)
Implant failures at LIV other than slip (%)	2 (5%)	0 (0%)	–	2 (4%)

In case of ARS, the mean magnitude of rod slip was 3.6 ± 2.2 mm, again with remarkable difference between the centers (Table 3). In half of the patients, rod slip was observed before 4 months. Slip was mostly observed on the convexity of the reduced distal curve. On that side, the residual rod length shortened, due to the preload that was used for correction and the residual load in case of remaining obliquity of the LIV (Fig. 4). Based on the preoperative curve configuration, the direction of slip could be predicted in 90% of the ARS cases. The remaining 10% were mainly bilateral slips that were often in the same direction (Fig. 4, case 2). To determine if anchor configuration was an important factor in the low-density constructs, the cases of slip in a single screw were compared to ARS cases with multiple distal screws on that side (Fig. 1 and Table 3). ARS rate appeared to be equally for both configurations.

**Table 3:** Characteristics of axial rod slips. sd = standard deviation

	Center 1 (* = 27 patients, ^†^ = 34 slips)	Center 2 (* = 18 patients ^†^ = 21 slips)	Center 3 (* = 11 patients ^†^ = 13 slips)	Total (* = 56 patients, ^†^ = 68 slips)
Slip distance at 1 year in mm (mean ± sd)	4.3 ± 3.1	3.9 ± 1.7	1.7 ± 0.6	3.6 ± 2.2
*n* per level of slip *(T11; T12; L1; L2; L3; L4)*	0; 7; 4; 0; 22; 1	1; 1; 3; 1; 10; 5	1; 2; 5; 1; 0; 4	1; 10; 12; 2; 32; 10
Slip within 4 months (%)*	14 (52%)	4 (22%)	5 (45%)	23 (41%)
Slip within 12 months (%)*	27 (100%)	18 (100%)	11 (100%)	56 (100%)
Unilateral, Convex slip*	17 (63%)	12 (67%)	7 (64%)	36 (64%)
Unilateral, concave slip*	3 (11%)	3 (17%)	2 (20%)	8 (14%)
Bilateral slip*	7 (26%)	3 (17%)	2 (20%)	12 (21%)
On single screw side (%)^†^	17 (50%)	7 (33%)	5 (38%)	29 (43%)
On double screw side (%)^†^	17 (50%)	14 (67%)	8 (62%)	39 (57%)
Concordance with expected direction (%)^†^	31 (91%)	17 (81%)	11 (92%)	59 (87%)

For * percentages are shown per total number of patients, for ^†^ per total number of slipped screws

Based on the above observations on slip direction that indicate that ARS is a consequence of overloading of the screw–rod interface, we selected potential risk factors: age, weight, percentage curve correction, LIV, and anchor density (Table 4). Weight and percent correction do not seem to influence the risk. However, an instrumentation including the caudal curve (LIV L3 or L4) was four times more often found in the patients with slip as compared to an LIV between T11 and L1.

**Table 4:** Characteristics of patients with and without axial rod slips

	With axial rod slip (*n* = 56)	Without axial rod slip (*n* = 148)
Age in years	15.3 ± 1.5	15.5 ± 2.5
Weight	63 ± 12	56 ± 11
% Coronal curve correction	56 ± 13	60 ± 11
Lumbar:thoracolumbar ratio*	1.9	0.5

*For this ratio, the patients with an LIV at L3 or L4 were compared to T11-L1

All cases of axial rod slip were communicated to the manufacturer to be reported in the MAUDE database.

## Discussion

This quality research paper describes the results of a systematic analysis to identify and better understand axial rod slip at the caudal end of the instrumentation in scoliosis surgery. The phenomenon of rod slip was recognized after  > 500 cases had been done, when confronted with several revision cases due to dislodgement of the rod. This failure to recognize subtle but sometimes clinically relevant rod slip on postoperative radiographs in daily practice appeared universal, as the surgeons of the other clinics also were unaware of this phenomenon in their cohorts until detailed inspection. Especially, when the slip distance is only millimeters, the resultant loss of correction is often overlooked or mistaken for adding on, or as a result of other mechanisms like crankshafting. The subtle changes that require magnification of the postoperative radiographs and similarity to adding on, probably explain the almost absence of literature and the very low reporting rate in the MAUDE database [10, 12]. Nevertheless, severe loss of correction and other failures can occur, which was a reason for us to convert to another end-of-construct screw until the magnitude of the problem and/or risk factors were identified.

Slippage of the CoCr rod in the locked screw head appeared to occur much more frequently than expected and almost always at the distal end of the construct where the axial load is largest. Therefore, we assume that the phenomenon itself is not so remarkable, as it simply shows overloading of the (grip) strength of this specific screw–rod connection. What is remarkable is that this limitation was observed in half of the cases before 4 months, a timeframe that the construct should be stable to allow for osseous fusion. If osseous fusion had failed after 1 year, fatigue could be a reason, but at this timepoint we did not observe the other signs of fatigue such as screw loosening or rod breakage. Apparently, the grip strength is relatively weak in comparison to the other mechanical features of this screw. With this failure mechanism in mind, we tried to identify objective risk factors such as patient weight and percent curve correction. We also looked at technical details that can be adapted (such as screw density and distal anchor configuration) without compromising the surgical aim to restore spinal balance and maximally correct in 3D, with minimal segments fused. Unfortunately, none of these factors investigated were really helpful to explain the occurrence and variance in occurrence of ARS among the centers. Curve correction and patient weight were not extraordinary for the slip cases and screw density at the LIV and LIV-1 was not decisive. There was, however, some variation in caudal anchor screw density between the centers. Therefore, definitive conclusions on the role of anchor configurations cannot be drawn. The most obvious factor, however, appears to be instrumentation of the (thoraco)lumbar curve, where the LIV is likely more forcefully corrected to save mobile lumbar segments.

We realize that this study has serious limitations and is not at all exhaustive to really identify the cause of the rod slip in these centers. First, we used 2D radiographs which cannot represent the exact length measurement and measurements are influenced by variations in body position. To address this problem, we determined the concordance between expected and measured slip direction, which was very high. Furthermore, on simulations of 5 and 10 degree body rotation in the axial and sagittal plane of 3D computed tomography scans of a postoperative scoliosis patient in this study, the effect on residual rod length measurements on 2D anterior–posterior digitally reconstructed radiographs was negligible. It should also be realized that the discrepancy in 2D and 3D length follows a sinusoidal pattern with little discrepancy with position change when the rod is close to vertical. Second, the length measurement was based on calibration of the rod which is a manual process. We recommend that this would involve a measurement error which we mitigated with multiple observers and a threshold of 1 mm. Also the interobserver reliability was very high. Therefore, we do not believe that there is a high risk of bias introduced by the applied measurement method. Third, the identification of curve-related risk factors was limited to the coronal plane and did not address the sagittal plane which very likely is of importance to spinal loading. We did not include that in the current study, because a harmonious sagittal spinal profile was always the goal and this is not likely a factor that influences the asymmetry in slip direction and it should not be changed to such an extent that it can prevent rod slip. Fourth, cases with alternative end-of-construct screws in center 3 were not included in this study. Center 3 changed in 2022 to another type of alternative screw (Everest, Stryker Corporation, Kalamazoo, MI, USA). Those data are not robust enough for comparison and further investigations will be needed to draw conclusions on alternative screws.

In the current study, three scoliosis centers in one country were involved; this might still be a reason to assume that rod slip is a local problem only. For example, we almost exclusively used CoCr rods instead of Titanium, and we never adopted the full-implant density approach as used to be popular in North America [13]. Therefore, further research is needed to identify the impact of ARS globally. Unfortunately, the resources to extend this study to other countries representing other techniques were not available to us.

## Conclusions

This study demonstrates the high prevalence of axial rod slip with the Mesa 2 Deformity System as used in the described setting. While minimal ARS can be subclinical, significant ARS should not be mistaken for adding on. To mitigate the problem of ARS, either less forceful correction should be performed or another type of (end-of-construct) screw should be considered. Given the fact that in scoliosis surgery, very different screw features are demanded for different locations, which is the reason for uniplanar and polyaxial screws, the idea of a one-size-fits-all deformity screw should be reconsidered. To better understand the relevance of the observed screw–rod interface failures, future research and post-market surveillance of axial rod slip occurrence with other surgical strategies are essential.

## Data Availability

Patient data is part of the standard patient record. Anonymized data on slip occurrence is stored at the research folder of the department > 15 year.
